# Exosomes as a messager to regulate the crosstalk between macrophages and cardiomyocytes under hypoxia conditions

**DOI:** 10.1111/jcmm.17162

**Published:** 2022-01-28

**Authors:** Zenglei Zhang, Yanyan Xu, Chang Cao, Bo Wang, Jiacheng Guo, Zhen Qin, Yongzheng Lu, Jianchao Zhang, Li Zhang, Wei Wang, Jinying Zhang, Junnan Tang

**Affiliations:** ^1^ Department of Cardiology The First Affiliated Hospital of Zhengzhou University Zhengzhou China; ^2^ Henan Province Key Laboratory of Cardiac Injury and Repair Zhengzhou China; ^3^ Henan Province Clinical Research Center for Cardiovascular Diseases Zhengzhou Henan China; ^4^ Henan Medical Association Zhengzhou China

**Keywords:** cardiomyocytes, cell‐cell interactions, exosomes, hypoxia, macrophage polarization

## Abstract

Recent studies have confirmed that cardiomyocyte‐derived exosomes have many pivotal biological functions, like influencing the progress of coronary artery disease via modulating macrophage phenotypes. However, the mechanisms underlying the crosstalk between cardiomyocytes and macrophages have not been fully characterized. Hence, this study aimed to observe the interaction between cardiomyocytes under hypoxia and macrophages through exosome communication and further evaluate the ability of exosomes derived from cardiomyocytes cultured under hypoxic conditions (Hypo‐Exo) to polarize macrophages, and the effect of alternatively activated macrophages (M2) on hypoxic cardiomyocytes. Our results revealed that hypoxia facilitated the production of transforming growth factor‐beta (TGF‐β) in H9c2 cell‐derived exosomes. Moreover, exosomes derived from cardiomyocytes cultured under normal conditions (Nor‐Exo) and Hypo‐Exo could induce RAW264.7 cells into classically activated macrophages (M1) and M2 macrophages respectively. Likewise, macrophage activation was induced by circulating exosomes isolated from normal human controls (hNor‐Exo) or patients with acute myocardial infarction (hAMI‐Exo). Thus, our findings support that the profiles of hAMI‐Exo have been changed, which could regulate the polarization of macrophages and subsequently the polarized M2 macrophages reduced the apoptosis of cardiomyocytes in return. Based on our findings, we speculate that exosomes have emerged as important inflammatory response modulators regulating cardiac oxidative stress injury.

## 1 INTRODUCTION

Myocardial infarction (MI) occurs due to a mismatch of oxygen and substrate supply in cardiomyocytes, which results in myocardial ischaemia and ultimately cell death.^1^ Despite advances in disease prevention, diagnosis and treatment, ischaemic heart diseases remain the primary cause of death worldwide.^2^, ^3^ The increasing morbidity of ischaemic heart failure has become a global burden,^4^ highlighting the urgent need for novel therapeutic strategies to repair damaged cardiomyocytes and alleviate pathological remodelling of the postischaemic heart.

Acute myocardial infarction arising from obstruction of the coronary circulation rapidly recruits an abundance of immune cells into ischaemic sites, leading to the release of high levels of inflammatory cytokines.^5^ The heart contains a population of macrophages^6^ that can rapidly expand after ischaemic injury due to the rapid recruitment of circulating monocytes that are mobilized from the bone marrow or spleen.^7^, ^8^, ^9^ Previous studies have revealed that macrophages play a significant role in the progress, pathology and repair of myocardial infarction.^7^, ^8^ The secretion of pro‐inflammatory media following cardiomyocytes injury leads to the recruitment of monocytes to the damaged sites.^10^, ^11^ In the dynamic post‐MI environment, the macrophage phenotypes may be tightly regulated by various cytokines and matricellular proteins, ultimately leading to sequential generation of macrophage subsets with distinct functional properties.^12^ Macrophages can be divided into two sequential phases. In the first phase, which peaks at day 3 after the onset of MI, pro‐inflammatory M1‐type macrophages accumulate around the infarcted myocardium to phagocytose dead cells and matrix debris.^13^, ^14^, ^15^ In the second phase, which occurs on day 6 after infarction onset, an increase in anti‐inflammatory M2‐type macrophages supervenes and promotes the repair of tissues through the release of anti‐inflammatory cytokines.^14^, ^15^

Exosomes are membrane‐encased vesicles ranging from 40 to 160 nm in size, and are released by prokaryotic and eukaryotic cells.^16^, ^17^ Exosomes have gained increased attentions in recent years as a result of their biological functions in intercellular communication, signalling and immune response regulating, as well as their potential abilities in diagnosing diseases and delivering drugs.^18^, ^19^, ^20^, ^21^ Previous studies have suggested that exosomes have major biological roles in regulating intercellular communication^17^ via their cargos, which include aqueous, harbouring DNA, microRNAs, proteins and lipids. Hypoxic cardiomyocyte‐derived exosomes can activate naïve macrophages into the M2 subtype, which is involved in the progression and resolution of post‐MI.^22^, ^23^ Nevertheless, the mechanisms underlying the crosstalk between cardiomyocytes and macrophages remain elusive and need to be further explored.

The present study demonstrates that cardiomyocyte‐derived exosomes can modulate macrophage polarization, and that polarized M2 macrophages can alleviate hypoxia‐induced cardiomyocytes injury. To our knowledge, this is the first study to confirm that the expression of TGF‐β increased in Hypo‐Exo. And Hypo‐Exo could induce RAW264.7 cells into M2 macrophages, which was further demonstrated by hAMI‐Exo. In return, M2 macrophages alleviated oxidative stress injury of H9c2 cells. Our results show that Hypo‐Exo or hAMI‐Exo may be a novel and promising therapeutic strategy for the recovery of injured cardiomyocytes via modulating the timely activation of M2 macrophages.

## 2 MATERIALS AND METHODS

### 2.1 Experimental animals and procedure

Animal protocols were approved by the experimental animal centre of Zhengzhou University. C57BL/6 mice (8‐week‐old male, 18–22 g) were purchased from Zhengzhou University (Zhengzhou, China). The mice were housed in the Animal Experiment Center in Zhengzhou University (Zhengzhou, China), serviced with a 12:12‐h light‐dark cycle, and provided a normal diet and purified water *ad libitum*. Animals were acclimatized to the laboratory environment for at least 1 week prior to the start of the experiment.

After intraperitoneal injection of pentobarbital for general anaesthesia, mice received endotracheal intubation and were artificially ventilated with room air. The chest was opened and the heart was exposed. The mice in the control, hNor‐Exo and hAMI‐Exo groups were intramyocardially injected with a suspension containing 50 μL phosphate‐buffered saline (PBS; Biological Industries), 300 μg of hNor‐Exo in 50 μL PBS and 300 μg of hAMI‐Exo in 50 μL PBS. The suspensions were injected at four sites in the largest cross section of the heart.

### 2.2 Immunohistochemistry staining

After 48 h, the mice were sacrificed and the hearts were immediately excised. A 0.9% sodium chloride solution was used to perfuse the hearts. The hearts were fixed in 4% paraformaldehyde for 24 h at room temperature, before dehydrating by immersing tissues through graded ethanol, embedding in paraffin and cutting horizontally into 4‐μm slices. To observe the inflammatory environment in the cardiac tissue, rabbit anti‐iNOS (ab15323; Abcam, Cambridge, MA, USA), and rabbit anti‐CD206 (ab64693; Abcam, Cambridge, MA, USA) were used to detect inflammation. The sections were incubated with the above fluorescent dye‐conjugated secondary antibodies and the nuclei were counterstained with 4′,6‐diamidino‐2‐phenylindole (DAPI).

### 2.3 Cell culture

H9c2 cells were purchased from the Cell Resource Center of Shanghai Institute and maintained in DMEM high glucose medium with 10% fetal bovine serum (FBS; Biological Industries, Israel) in 5% CO_2_ at 37°C. Then, conditioned medium was collected for exosome isolation. Briefly, H9c2 cells were seeded into T‐75 flasks. When cultures reached approximately 90% confluency, the cells were washed thrice with PBS and blanked for 24 h with FBS‐free DMEM high glucose medium, followed by culturing for another 48 h with 8 mL FBS‐free DMEM high glucose medium under normal conditions or hypoxic conditions (<1% O_2_) until collection of the conditioned medium for exosome isolation.

RAW264.7 cells were obtained from the Cell Resource Center of Shanghai Institute and maintained under the same conditions. To polarize M1 and M2 macrophages, RAW264.7 cells were cocultured with LPS (100 ng/mL, Sigma‐Aldrich, USA) or IL‐4 (40 ng/mL, BD, USA), respectively, for 24 h.

### 2.4 Isolation of exosomes from cultured cells

ExoQuick‐TC Exosome Precipitation Solution (System Biosciences, USA) was used to extract exosomes from the conditioned medium of cells according to the manufacturer's instructions. Briefly, culture supernatants were collected and spun in a Centrifuge 5810R (Eppendorf, Germany) at 3000 *g* for 15 min at 4°C to remove cells and debris. Then, 40 mL cell supernatants were mixed with 8 mL ExoQuick‐TC, mixed well and refrigerated overnight (at least 12 h). The ExoQuick‐TC/supernatant mixture was centrifuged at 1500 *g* for 30 min and 1500 *g* for 5 min at 4°C to collect the exosomes. Next, the pelleted exosomes were concentrated to 10^10^/mL by resuspending in PBS and stored at −80°C for further use after quantification using a BCA Protein Assay Kit (PC0020; Solarbio, Beijing, China) (the concentration of protein was about 0.5 μg/μL).

### 2.5 Circulating exosome isolation from plasma

All of the patients were recruited from the First Affiliated Hospital of Zhengzhou University and Nanyang central hospital, and samples were obtained according to the Declaration of Helsinki (2008), and approved by the Ethic Committee of First Affiliated Hospital of Zhengzhou University. And all the enrolled patients had signed the informed consent form. The basic clinical data for these patients are listed in Table S1. AMI was diagnosed according to the 2017 guidelines released by the European Society of Cardiology.^24^ Plasma samples were collected from AMI patients within 24 h of admission. Healthy patients were enrolled as controls. After centrifuging blood samples at 3000 *g* for 10 min at 4°C, the 250 μL supernatant was collected to obtain exosomes via adding 63 μL ExoQuick reagent (System Biosciences, USA) and incubated for 1 h at 4°C. After centrifugation at 1500 *g* for 30 min and 1500 *g* for 5 min at 4°C, the pelleted exosomes were concentrated to 10^12^/mL by resuspending in PBS, and the exosome solution was stored at –80°C after quantification using a BCA Protein Assay Kit (the concentration of protein was about 45 μg/μL).

### 2.6 miRNA library construction and sequencing

Circulating exosomes from AMI patients (*n* = 6) and healthy control (*n* = 3) were used to perform miRNA sequencing to identify the miRNA profiles of Nor‐Exo and AMI‐Exo by the Illumina HiSeq 2500 platform, which was conducted by a commercial service (Genesky Biotechnologies, China).

### 2.7 Transmission electron microscopy (TEM)

TEM (Tecnai G2 spirit Bio TWIN) was conducted to assess the morphology of the exosomes as previously described.^25^ A drop of exosome pellets (20 μL) was adsorbed onto 200‐mesh carbon‐coated copper grids, and any excess fluid was blot‐dried by filter paper. After incubating for 5 min at room temperature and air‐drying, the exosomes were examined by TEM.

### 2.8 Nanoparticle tracking analysis

Exosome preparations were diluted with PBS to gain the appropriate detection concentration, and nanoparticle tracking analysis (NTA, ZetaView PMX 110, Particle Metrix, Meerbusch, Germany) was used to measure the size distribution.

### 2.9 Uptake of exosomes by RAW264.7 cells

Exosomes was labelled with a PKH26 kit (PKH26 Red Fluorescent Cell Linker Kit, Sigma, USA) following the manufacturer's instructions. In detail, 200 μL Solution C was added to 100 μL of exosomes suspended in PBS. Another 200 μL of Solution C containing 1 μL of PKH26 dye were mixed with the above solution and incubated for 5 min at room temperature, and then the reaction was stopped by adding 400 μL of a 5% BSA solution. Exosomes labelled with PKH26 were isolated using ExoQuick‐TC. Next, 100 μL of PKH26‐labelled exosomes were diluted in 400 μL DMEM and incubated with RAW264.7 cells for 4 h.

To determine inhibition of exosomes taken up by RAW264.7 cells, the PKH26‐labelled exosomes were cocultured with RAW264.7 cells for 4 h after pretreatment with Dynasore (80 μmol/L) for 30 min, followed with DAPI (1:1000) staining at room temperature for 20 min. After washing with PBS thrice, a Confocal Laser Scanning Microscope (Nikon, A1 + R, Tokyo, Japan) was used to observe exosomes taken up by RAW264.7 cells.

### 2.10 Different sourced exosomes treatment on macrophage

To reveal the effect of different sourced exosomes on polarization of naïve macrophage, exosomes derived from H9c2 cells cultured under normal conditions (Nor‐Exo) or H9c2 cells cultured under hypoxic conditions (Hypo‐Exo) and exosomes isolated from the plasma of human healthy controls (hNor‐Exo) or acute myocardial infarction patients (hAMI‐Exo) were cocultured with RAW264.7 macrophages in vitro. Briefly, RAW264.7 cells were seeded into 6‐well plates for a maximum of five passages. After cell adhesion, the cells were treated with exosome‐free medium containing PBS, Nor‐Exo (100 μg/mL), or Hypo‐Exo (100 μg/mL) for 48 h. To further detect the effect of circulating exosomes on modulating naïve macrophage polarization, hNor‐Exo or hAMI‐Exo were cocultured with RAW264.7 cells as described above.

### 2.11 Western blot analysis

Western blotting was performed to identify the exosome markers CD63 (ab108950/ab13405; Abcam, USA), HSP70 (ab2787; Abcam) and Alix (92880; Cell Signaling Technology, USA), and to determine the protein expression levels of TGF‐β (ab92486; Abcam), HIF‐1α (ab179483; Abcam), iNOS (ab178945; Abcam), Arg‐1 (ab91279; Abcam), Bax (ab182733; Abcam), Bcl‐2 (3498; Cell Signaling Technology), GAPDH(ab8245; Abcam) and tubulin (ab7291; Abcam). Briefly, RIPA lysis buffer (Solarbio, China) was used to lyse cells and exosomes. Then, the samples were mixed with loading buffer, heated at 95°C, loaded on SDS‐polyacrylamide gels for electrophoresis, and finally transferred onto PVDF membranes (Millipore, USA). The membranes were blocked with 5% nonfat milk (BD, USA) and then incubated with the following primary antibodies at 4°C overnight: anti‐CD63(1:1000), anti‐HSP70 (1:1000), anti‐Alix (1:1000), anti‐GAPDH (1:1000), anti‐TGF‐β (1:1000), anti‐HIF‐1α (1:1000), anti‐iNOS (1:1000), anti‐Arg‐1 (1:1000), anti‐Bax (1:1000), anti‐Bcl‐2(1:1000), and anti‐tubulin (1:5000). Before incubating with secondary antibodies (1:10,000) for 1.5 h at room temperature, the membranes were carefully washed with TBST. A horseradish peroxidase kit (Thermo Fisher Scientific, USA) and Amersham Imager 600 (GE Healthcare Life Sciences, USA) were used to detect and capture the proteins. All protein expression levels were assessed by three independently repeated experiments and analysed using ImageJ software (NIH, USA).

### 2.12 Transwell assays

To evaluate whether polarized macrophages could alleviate oxidative stress injury of H9c2 cells, RAW264.7 cells were cocultured with PBS, LPS (100 ng/mL, Sigma‐Aldrich, USA), or IL‐4 (40 ng/mL, BD, USA) for 24 h on the upper chamber. Then, the upper chamber was moved into the Transwell system (six‐well plate, Corning, New York, NY, USA) for 48 h, where the lower chamber was seeded with H9c2 cells. Next, the H9c2 cells were treated by 100 μmol/L H_2_O_2_ for 4 h.

### 2.13 Quantitative RT‐PCR analysis

To evaluate the level of inflammatory cytokine gene expression in vitro, RAW264.7 cells stimulated by differently sourced exosomes were harvested, and total RNA was extracted using TRIzol reagent (Thermo Fisher Scientific, USA). Then, the cytokine expression (IL‐1β, IL‐6, iNOS, TNF‐α, MCP‐1, Arg‐1, TGF‐β, IL‐10, Ym‐1 and Fizz‐1) was determined using qRT‐PCR. And the sequences of primers used for qRT‐PCR analysis are presented in Table S2.

### 2.14 TUNEL assay

According to the manufacturer, TUNEL assay (Roche Applied Science, Indianapolis, IN, USA) was conducted to detect apoptosis of H9c2 cells. Briefly, H9c2 cells (bottom chamber) and M1 or M2 macrophages (upper chamber) were cocultured in Transwell systems with 0.4‐μm pore size for 48 h (24‐well Corning). Then, the H9c2 cells were treated with 100 μmol/L H_2_O_2_ for 4 h. After fixing with 4% paraformaldehyde, the cells were stained with 100 μL of the TUNEL reaction mixture (Roche Applied Science, Indianapolis, IN, USA) for 60 min at 37°C. Nuclei were stained with DAPI (1:1000). Finally, the cells were visualized under a Confocal Laser Scanning Microscope (Nikon, A1 + R, Tokyo, Japan).

### 2.15 Statistical analysis

The data are reported as the mean ± SD. The normal distribution and the homoscedasticity of the data were tested using the D’Agostino's‐Pearson normality test and the Bartlett's test, respectively. Two group comparisons and multiple group comparisons were performed by 2‐tailed unpaired Student's *t* test and 1‐way ANOVA followed by Bonferroni post hoc correction respectively. All of the statistical analyses were conducted using GraphPad Prism 8.0 (GraphPad Software, Inc., San Diego, CA, USA). *p*‐Values < 0.05 were considered to be significant.

## 3 RESULT

### 3.1 Characterization and uptake of exosomes derived from cardiomyocytes

**FIGURE 1:**
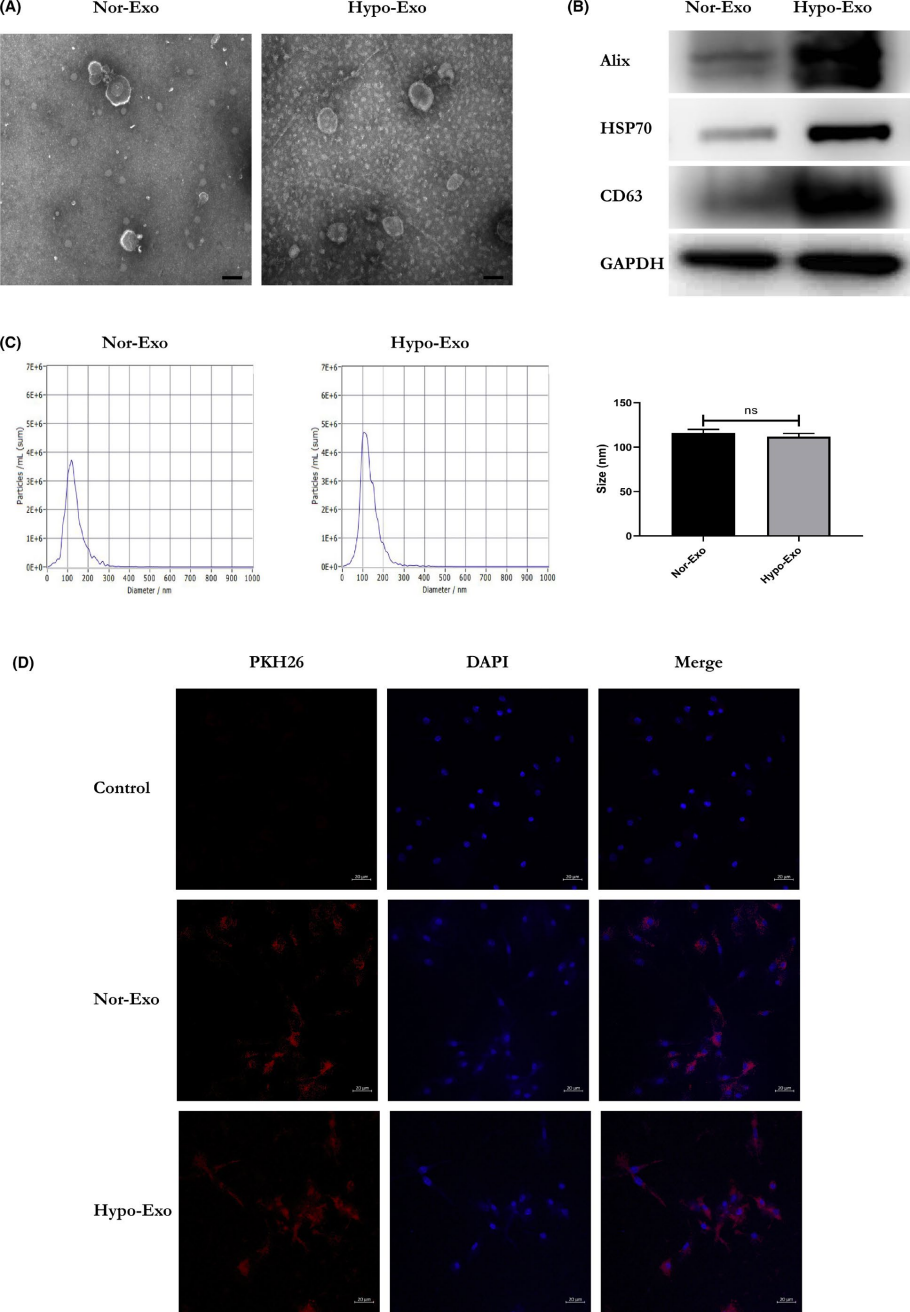
The characterization and uptake of Exo derived from H9c2 cell lines cultured in normoxic or hypoxic environment. (A) Morphological characterization of Nor‐Exo and Hypo‐Exo by transmission electron microscopy (scale bar = 100 nm). (B) The surface makers (Alix, HSP70 and CD63) of Nor‐Exo and Hypo‐Exo were detected by western blotting. (C) The size distribution was measured using NanoSight analysis, which indicated that the diameter of most of the particles was within the range of 40–160 nm. ‘ns’ indicates *p* ≥ 0.05. (D) A laser scanning confocal microscope captured the PKH26‐labelled Exos (red fluorescence) taken up by RAW264.7 cells stained by DAPI (blue fluorescence) (scale bar = 20 μm)

To obtain Nor‐Exo and Hypo‐Exo, cardiomyocytes were cultured under normal conditions or hypoxic conditions for 48 h. Nor‐Exo and Hypo‐Exo were extracted from the conditioned media, and TEM was performed to assess the morphology of Nor‐Exo and Hypo‐Exo, by showing presence of round, cup‐shaped morphology (Figure 1A). The specific exosome markers (Alix, HSP70 and CD63) were detected as shown in Figure 1B. Meanwhile, NTA revealed that the vesicles size distribution was within the range of 40–160 nm (Figure 1C).

To confirm whether macrophages could internalize cardiomyocyte‐derived exosomes, the isolated exosomes were labelled with PKH26 (red fluorescence), a fluorescent membrane dye. After incubation with PKH26‐labelled exosomes for 4 h, we assessed the presence of fluorescently labelled exosomes within macrophages. The results implied that both Nor‐Exo and Hypo‐Exo were internalized by macrophages (Figure 1D).

### 3.2 Cardiomyocyte‐derived exosomes could modulate the activation profiles of macrophages in vitro

**FIGURE 2:**
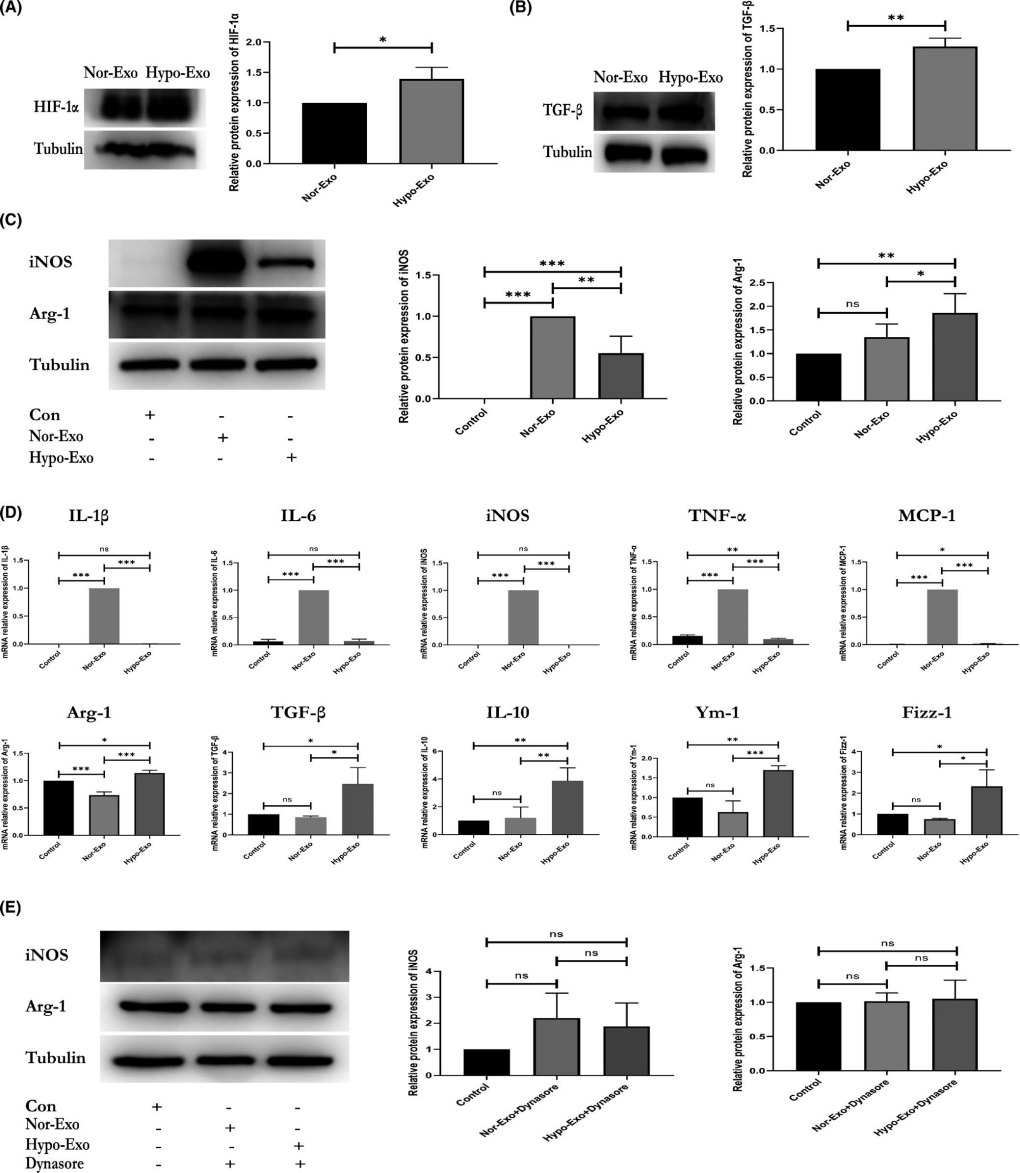
Nor‐Exo and Hypo‐Exo derived from H9c2‐polarized macrophages. (A) Quantitative analysis of hypoxia‐inducible factor 1 alpha expression in Nor‐Exo and Hypo‐Exo as detected by western blotting. (B) Quantitative analysis of transforming growth factor beta expression in Nor‐Exo and Hypo‐Exo as detected by western blotting. (C) M1‐macrophage marker iNOS and M2‐macrophage marker Arg‐1 were evaluated in RAW 264.7 cells cocultured with PBS, Nor‐Exo or Hypo‐Exo for 48 h and assessed by western blotting. (D) The mRNA expression levels of IL‐1β, IL‐6, iNOS, TNF‐α, MCP‐1, Arg‐1, TGF‐β, IL‐10, Ym‐1 and Fizz‐1 in RAW 264.7 cells were determined by qRT‐PCR. (E) Western blot analysis of Arg‐1 levels in RAW264.7 cells pretreated with or without Dynasore, followed by the stimulation with PBS, Nor‐Exo or Hypo‐Exo. At least three independent experiments were performed for each group. Data are expressed as the mean ± SD, *p < 0.05, **p < 0.01, ***p < 0.001; ‘ns’ indicates *p* ≥ 0.05

It has been reported that exosomes released by cardiomyocytes during ischaemia transform macrophages into M2‐type macrophages, which are involved in cardiac repair.^22^ However, the mechanisms underlying the crosstalk between cardiomyocyte‐derived exosomes and macrophages remain elusive. Moreover, a recent study found that TGF‐β‐mediated Smad3 activation in M2‐type macrophages plays a critical role in cardiac repair.^12^ Nevertheless, it is still uncertain that whether Hypo‐Exo induces macrophage polarization toward the M2 phenotype by increasing the expression of TGF‐β. To ensure hypoxic model stabilization, the expression of hypoxia‐inducible factor‐1 alpha (HIF‐1α), a known hypoxic biomarker,^26^, ^27^ was measured in Nor‐Exo and Hypo‐Exo. The result showed that the HIF‐1α was highly expressed in Hypo‐Exo (Figure 2A). To make the result more reliable, we further detected the expression of HIF‐1α in H9c2 cells cultured under normal and hypoxic conditions. Concordant with the result obtained above, our results showed that the HIF‐1α was highly expressed in H9c2 cells cultured under hypoxic conditions (Figure S1). To detect the signalling pathway through which cardiomyocyte‐derived exosomes polarized macrophages, we detected the expression of TGF‐β between Nor‐Exo and Hypo‐Exo. As shown in Figure 2B, higher expression of TGF‐β was observed in the Hypo‐Exo compared to the Nor‐Exo.

Because AMI rapidly recruits immune cells into ischaemic sites,^5^ numerous studies have suggested that macrophages have a crucial role in the inflammatory response of post‐MI.^7^, ^8^, ^9^ Moreover, cardiomyocyte‐derived exosomes can modulate the activation profile of macrophages.^22^ To address this, we measured the expression level of inducible nitric oxide synthase (iNOS) and arginase‐1 (Arg‐1) (biomarkers of M1 and M2‐type macrophages respectively) in macrophages cocultured with Nor‐Exo or Hypo‐Exo. The result showed that Hypo‐Exo increased the expression of Arg‐1 and decreased the expression of iNOS (Figure 2C). However, the opposite result was observed in the Nor‐Exo treatment group (Figure 2C). We further investigated the genetic levels of activation profiles when macrophages were cocultured with Hypo‐Exo or Nor‐Exo by qRT‐PCR analysis. In Figure 2D, we observed a significant decrease in the production of M1 biomarkers IL‐1β, IL‐6, iNOS, TNF‐α and MCP‐1, and a remarkable increase in the production of M2 biomarkers Arg‐1, TGF‐β, IL‐10, Ym‐1, and Fizz‐1 in the Hypo‐Exo treatment group. In the Nor‐Exo treatment group, the expression of these biomarkers showed opposite results (Figure 2D).

To further detect the involvement on exosomes endocytosis upon macrophage response, macrophages were pretreated with Dynasore, an endocytic inhibitor, for 30 min, prior to incubation with PKH26‐labelled exosomes for 4 h. Figure S2 shows that exosome uptake by macrophages was inhibited by Dynasore. In addition, when macrophages were pretreated with Dynasore, followed by incubation with exosomes, no change in iNOS and Arg‐1 expression were observed 48 h after coculture (Figure 2E). Together, these results show that cardiomyocyte‐derived exosomes can modulate the activation profiles of macrophages, and Hypo‐Exo can induce macrophages to exhibit the M2 subtype.

### 3.3 Circulating exosomes regulate the activation profiles of macrophages in vitro and in vivo

**FIGURE 3:**
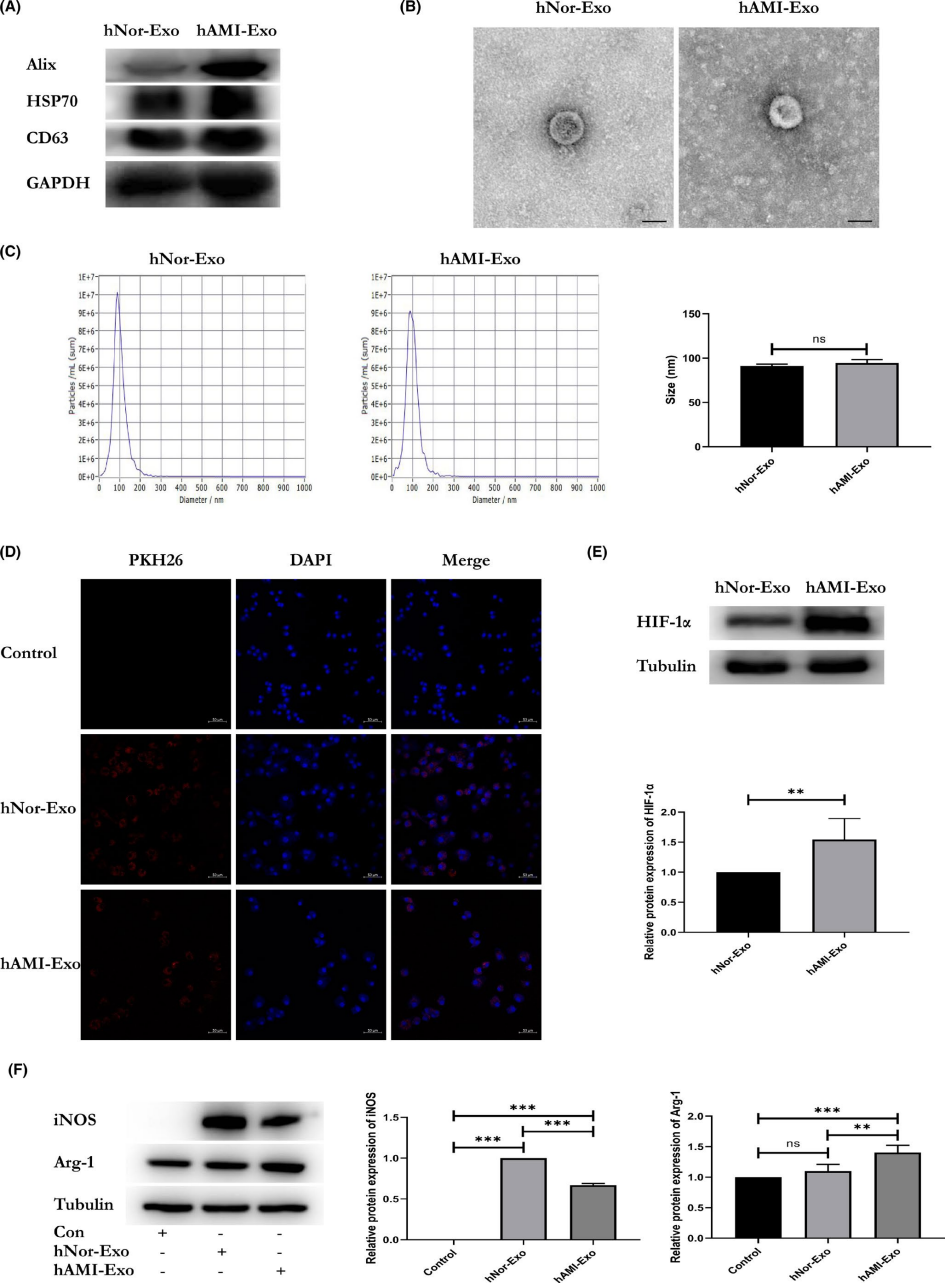
Circulating exosomes modulate macrophage polarization in vitro. (A) The protein profile of hNor‐Exo and hAMI‐Exo was evaluated by western blotting. (B) Morphological characterization of hNor‐Exo and hAMI‐Exo by transmission electron microscopy (scale bar = 50 nm). (C) The size distribution of hNor‐Exo and hAMI‐Exo was measured using Nanosight analysis. ‘ns’ indicates *p* > 0.05. (D) A laser scanning confocal microscope captured the PKH26‐labelled hNor‐Exo or hAMI‐Exo (red fluorescence) taken up by RAW264.7 cells stained by DAPI (blue fluorescence) (scale bar = 50 μm). (E) Western blot analysis of HIF‐1α expressed in hNor‐Exo or hAMI‐Exo (*n* = 3). (F) RAW 264.7 cells were stimulated with PBS, hNor‐Exo or hAMI‐Exo for 48 h, the M1‐macrophage marker iNOS and M2‐macrophage marker Arg‐1 were analysed by western blot (*n* = 3). Data are expressed as means ± SD, *p < 0.05, **p < 0.01, ***p < 0.001; “ns” indicates *p* ≥ 0.05

To translate the results to the human pathophysiological context, we further explored the effect of circulating exosomes derived from human plasma of patients with AMI (hAMI‐Exo) or normal controls (hNor‐Exo) on macrophage activation. First, western blotting was used to characterize the exosomes, showing that these were enriched with exosome markers, such as CD63, HSP70 and Alix (Figure 3A). The morphology and size of the circulating exosomes were measured via TEM (Figure 3B), and NTA was used to further evaluate the size distribution of exosomes (Figure 3C). The interactions between macrophages and circulating exosomes were evaluated, which showed that both hNor‐Exo and hAMI‐Exo were internalized by macrophages (Figure 3D).

Next, to further assess the exosomes derived from human plasma of patients with AMI, the biomarker of hypoxia HIF‐1α was detected, and the results revealed that HIF‐1α was highly expressed in hAMI‐Exo (Figure 3E). Moreover, we evaluated whether circulating exosomes could modulate the activation profiles of macrophages. Concordant with the results obtained with cardiomyocyte‐derived exosomes, our results demonstrated that hAMI‐Exo decreased iNOS expression and increased Arg‐1 expression compared with hNor‐Exo or the control group (Figure 3F).

**FIGURE 4:**
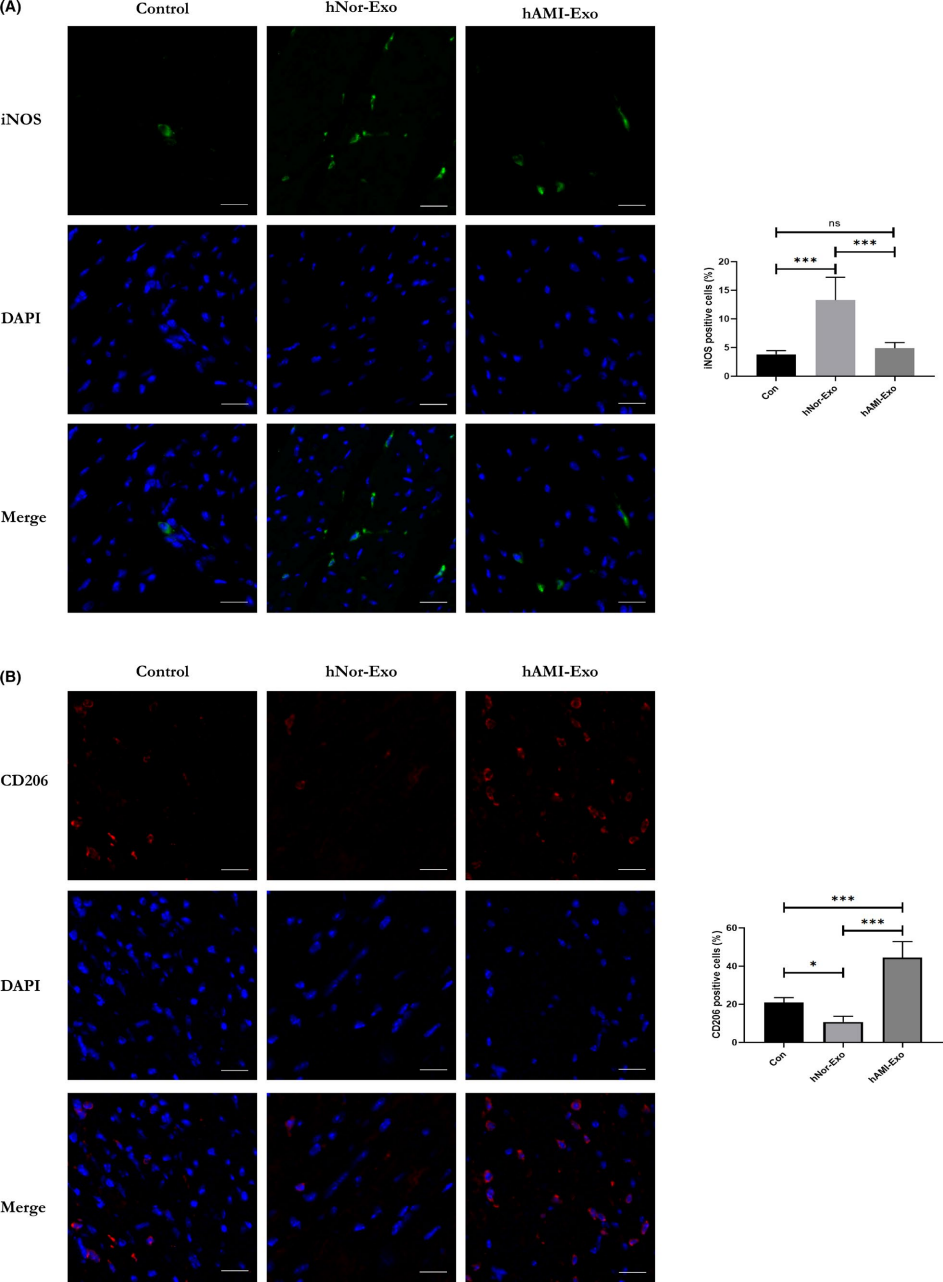
Circulating exosomes modulate macrophage polarization in vivo. (A) Representative images of iNOS‐positive staining: DAPI, blue; iNOS, green (*n* = 6, scale bar = 20 μm). (B) Representative images of CD206‐positive staining: DAPI, blue; CD206, red (*n* = 6, scale bar = 20 μm). Data are expressed as means ± SD, *p < 0.05, **p < 0.01, ***p < 0.001; ‘ns’ indicates *p* ≥ 0.05

To further evaluate the ability of hypoxic cardiomyocyte‐derived exosomes to induce M2 macrophage activation in cardiac tissue, hNor‐Exo and hAMI‐Exo were injected into the myocardium. The results showed that the hAMI‐Exo decreased iNOS expression (Figure 4A) and increased CD206 (Figure 4B) expression in cardiac tissue compared to hNor‐Exo or the control group, which is consistent with the in vitro data. Based on our results, hAMI‐Exo can also modulate M2 macrophage activation.

### 3.4 miRNAs might be the key role in regulating naïve macrophages polarization

**FIGURE 5:**
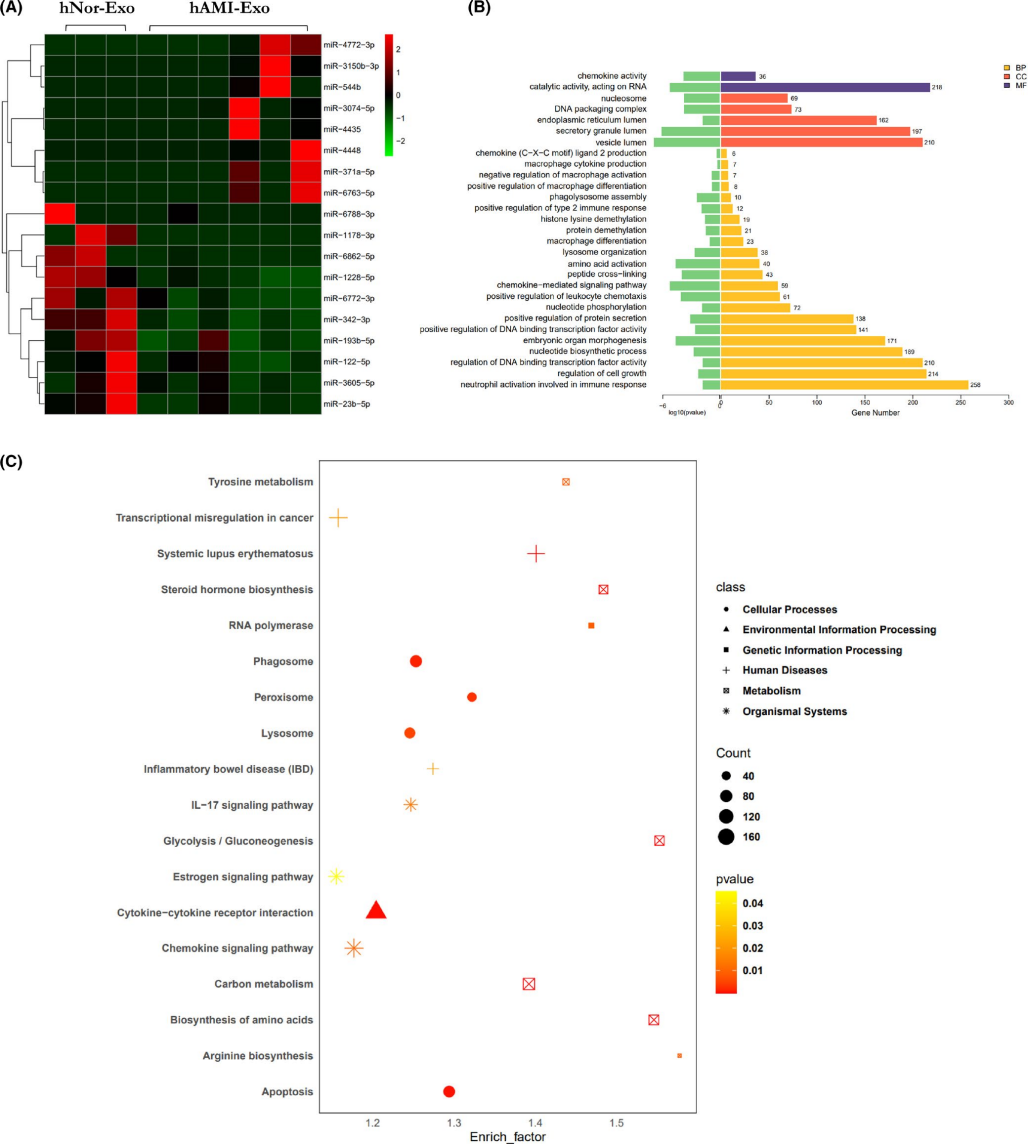
The related profiles and functions of miRNAs in circulating exosomes. (A) The heat map of differential expression microRNAs in exosomes derived from hNor‐Exo (*n* = 3) and hAMI‐Exo (*n* = 6). The red dots: up‐regulated microRNAs. Green dots: down‐regulated microRNAs. (B) GO analyses for target genes of the differentially expressed miRNAs. (C) KEGG pathway analyses of the target genes of the differentially expressed miRNAs

Both the Hypo‐Exo and hAMI‐Exo have showed the ability of inducing naïve macrophages into M2 subtype according to the mentioned above results. Mounting evidence suggests that the profiles of miRNAs may change in the hypoxic condition and play a vital role in modulating the polarization of macrophages.^28^, ^29^ To further explore the potential mechanisms, Illumina HiSeq 2500 high‐throughput sequencing was conducted to determine the miRNA expression profiles between the hNor‐Exo and hAMI‐Exo. And the 18 differently expressed microRNAs were found (Figure 5A). Next, to elucidate the potential role of miRNAs in hNor‐Exo and hAMI‐Exo, GO and KEGG analysis were performed. As shown in Figure 5B, these differently expressed microRNAs are mainly enriched in various biological process, such as chemokine (C‐X‐C motif) ligand 2 production (GO:0072567), macrophage cytokine production (GO:0010934), positive regulation of type 2 immune response (GO:0002830) and macrophage differentiation (GO:0030225). Target genes were mapped to KEGG pathways, and some representative pathways were shown in Figure 5C.

### 3.5 M2 macrophages alleviate oxidative stress injury in H9c2 cells in vitro

**FIGURE 6:**
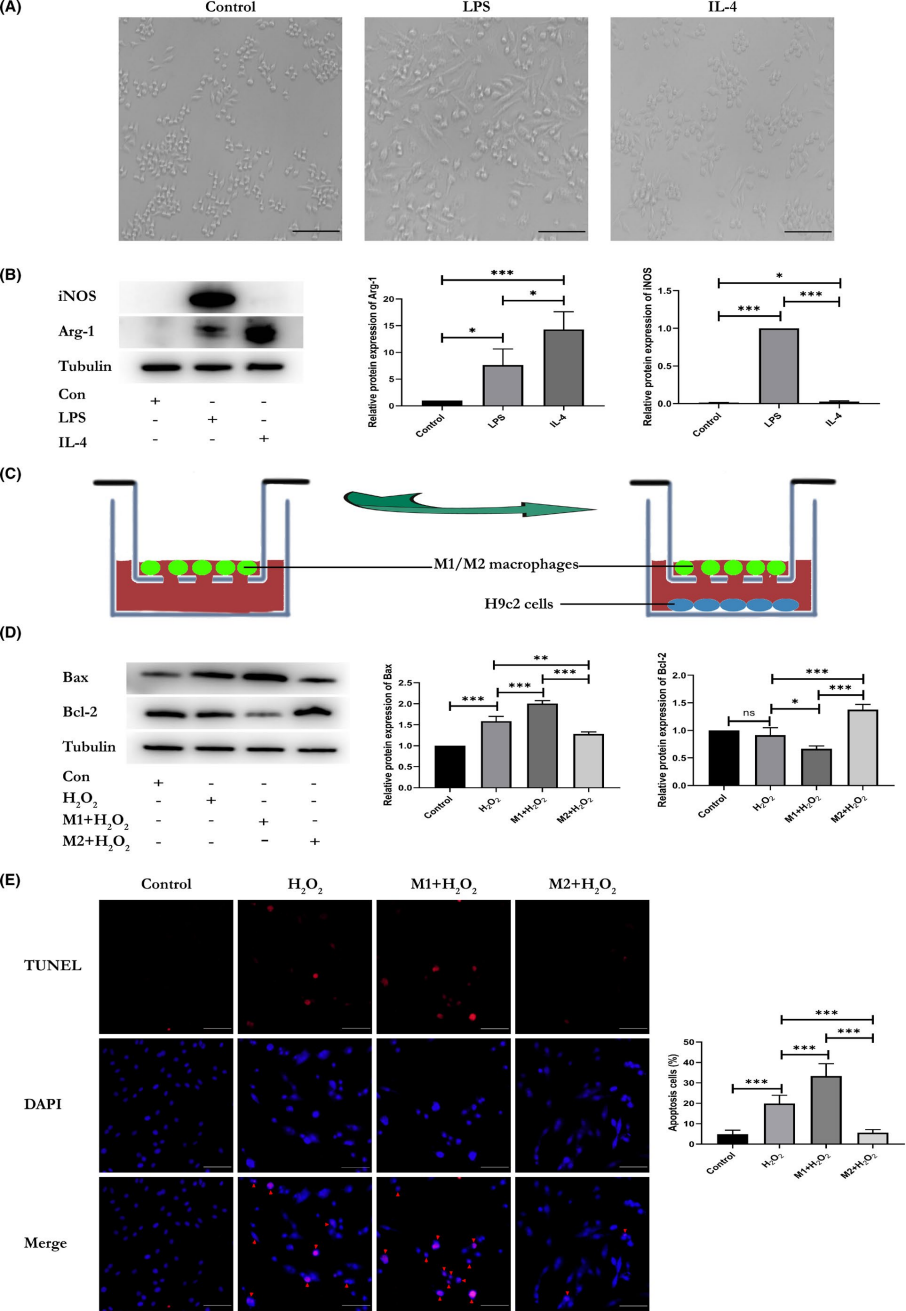
M2 macrophages alleviate oxidative stress injury in H9c2 cells. (A) Optical image of RAW264.7 cells treated with PBS, LPS (100 ng/mL) or IL‐4 (40 ng/mL) for 24 h. (B) Western blot analysis of iNOS and Arg‐1 expressed in RAW264.7 cells treated with PBS, LPS (100 ng/mL) or IL‐4 (40 ng/mL) for 24 h (*n* = 3). (C) Schematic image of H9c2 cells (bottom chamber) cocultured with M1 macrophages [RAW264.7 cells treated with LPS] or M2 macrophages [RAW264.7 cells treated with IL‐4] (upper chamber) in Transwell systems. (D) Expression of apoptosis‐related proteins (Bax and Bcl‐2) were evaluated in H_2_O_2_‐induced H9c2 cells cocultured with M1 macrophages or M2 macrophages and assessed by western blotting (*n* = 3). (E) Representative TUNEL staining for apoptotic cells in H_2_O_2_‐treated H9c2 cells cocultured with M1 macrophages or M2 macrophages; the red fluorescence represents apoptotic cells (*n* = 4, scale bar = 100 μm). Data are expressed as means ± SD, *p < 0.05, **p < 0.01, ***p < 0.001; ‘ns’ indicates *p* ≥ 0.05

Recent studies have focused on the plasticity of macrophages, which are considered as the therapeutic target of many diseases, particularly ischaemic heart disease and cancer.^30^, ^31^ In addition, numerous studies have suggested that M2 macrophages play a central role in responses to parasites, wound repair, angiogenesis and allergic diseases by secreting soluble chemokines, cytokines, growth factors and exosomes.^5^, ^32^, ^33^ To address the effect of macrophages on oxidative stress, we treated naïve macrophages with LPS (100 ng/mL) or IL‐4 (40 ng/mL) for 24 h to polarized them into the M1 or M2 phenotype. The morphology of M1 and M2 macrophages was captured by an inverted microscope (Figure 6A), and M1 and M2 macrophage were detected using biomarkers iNOS and Arg‐1 (Figure 6B).

Next, we also checked the expression of reactive oxygen species (ROS) in H_2_O_2_‐treated H9c2 cells (Figure S3). Then, the H9c2 cells (bottom chamber) and M1 or M2 macrophages (upper chamber) were cocultured in Transwell systems with a 0.4‐μm pore size for 48 h (Figure 6C), after which the H9c2 cells were treated with 100 μmol/L H_2_O_2_. After 4 h of coculture, apoptosis‐related marker proteins (Bax and Bcl‐2) were evaluated in H9c2 cells by western blotting. Decreased expression of Bax and increased expression of Bcl‐2 were observed in the M2 macrophages treatment group, which suggested that the M2 macrophages alleviate oxidative stress injury of H9c2 cells, while M1 macrophages worsen the injury (Figure 6D). We further evaluated the effect using a TUNEL assay and obtained similar results (Figure 6E). Taken together, these findings indicated severe anti‐oxidative stress injury in M2 macrophages group.

## 4 DISCUSSION

Our study further illustrates the interaction between cardiomyocytes and macrophages through exosomes under hypoxic conditions, which mimic the ischaemic environment after myocardial infarction. In this study, to further confirm that exosomes were derived from hypoxia‐induced cardiomyocytes or human serum of AMI patients, HIF‐1α, a hypoxia biomarker, was detected. The result suggested the expression of HIF‐1α increased in either Hypo‐Exo or hAMI‐Exo compared with Nor‐Exo or hNor‐Exo. This is concordant with a recent study that showed that after hypoxia exposure, HIF‐1α protein expression was remarkably upregulated.^27^

We also showed that the expression of TGF‐β of Hypo‐Exo increased compared with Nor‐Exo. In fact, previous works reported that cultured primary mouse hepatocytes secreted latent‐TGF‐β, which was activated when the hepatocytes were subjected to hypoxic conditions.^34^ Previous studies have suggested that cardiomyocyte‐derived exosomes can activate macrophages to get involved in the progression and resolution of post‐MI.^22^, ^23^ Moreover, a recent study by Chen et al. reported that TGF‐β has an indispensable role in repairing myocardial injury and promoting fibrosis by activating both Smad‐dependent and non‐Smad pathways.^12^ We further explored the effect of Hypo‐Exo or hAMI‐Exo on modulating macrophage activation. Our results showed that Hypo‐Exo or hAMI‐Exo increased the protein expression of Arg‐1 and decreased iNOS expression compared to Nor‐Exo and hNor‐Exo. Furthermore, similar results were confirmed at the genetic level by qRT‐PCR, showing a significant decrease in the production of M1 biomarkers and a remarkable increase in the production of M2 biomarkers. According to our data, hypoxia could alter the effects of cardiomyocyte‐derived exosomes on macrophage activation and induce macrophages into an anti‐inflammatory subset to alleviate oxidative stress injury of H9c2 cells. However, additional *in vivo* experiments are required to assess the overall effect of exosomes released by cardiomyocytes during ischaemia on disease outcome. And according to the results from high‐throughput sequencing, the miRNA expression profiles are significantly different between hNor‐Exo and hAMI‐Exo. The results suggested that miRNAs might play a vital role in modulating the polarization of macrophages, which is in accordance with the previous study.^29^

These results may have important clinical implications and raise some intriguing hypotheses. Indeed, it has been shown that Hypo‐Exo and hAMI‐Exo induce macrophages into an anti‐inflammatory type, potentially contributing to angiogenesis and postinfarction repair. In addition, emerging evidence has demonstrated that M2 macrophages critically facilitate the repair of infarcted heart via regulating fibroblast activation in a murine model,^35^ further supporting our findings. Thus, increased recruitment and infiltration of M2 macrophages by Hypo‐Exo and hAMI‐Exo after AMI may have favourable effects on infarct healing.

Moreover, we evaluated the effect of macrophages on cardiomyocytes under oxidative stress by building a M1 and M2 macrophages model. M2 macrophages effectively alleviated oxidative stress injury of cardiomyocytes, as indicated by the expression of apoptosis‐related marker proteins (Bax and Bcl‐2). Moreover, the results of the TUNEL assay showed that M2 macrophages significantly reduced apoptosis, while M1 macrophages worsened the oxidative stress injury. Our results are in line with those in a previous study^35^ that IL‐4‐induced macrophages potentiate the effects of myocardial repair by promoting capillary formation and formation of connective tissues in ischaemic areas. Although a large number of studies^36^, ^37^, ^38^, ^39^, ^40^ on myocardial repair have been reported, our results show that exosome derived from hypoxic cardiomyocytes may be another novel and promising therapeutic strategy for the recovery of injured myocardium via modulating the polarization profiles of macrophages.

## 5 LIMITATIONS

There are still limitations in the study. First, our results did not indicate which component(s) of exosomes play(s) a major role in mediating macrophage activation. Second, although some differently expressed microRNAs were found in the present study, we failed to identify and clarify the detailed mechanisms, and we only simply stated and prudently interpreted our findings instead of exaggerating them in order to be responsible for the conclusion. We are exploring the potential mechanisms of how these 18 differently expressed microRNAs between the hNor‐Exo and hAMI‐Exo modulate the naïve macrophages polarization, which must be a very meaningful work. And we hope more researchers join the work to make the work completer and more reliable. Furthermore, although we demonstrated that M2 macrophages induced by hNor‐Exo and hAMI‐Exo can alleviate oxidative stress injury in vitro, the therapeutic potentials of M2 macrophages in improving the cardiac function were not directly demonstrated considering the previous studies, and the specific components produced by macrophages, such as the soluble chemokines, cytokines, growth factors or exosomes, could be confirmed and thus require further investigation.

## 6 CONCLUSION

Our results demonstrated that the interaction between cardiomyocytes and macrophages is indispensable for maintaining heart homeostasis via inducing the polarization of macrophages through cardiomyocyte‐derived exosomes, and the exosomes have emerged as important inflammatory response modulators in regulating cardiac oxidative stress injury.

## CONFLICT OF INTEREST

The authors have no conflicts of interest to declare.

## AUTHOR CONTRIBUTION

**Zenglei Zhang:** Conceptualization (equal); Data curation (equal); Formal analysis (equal); Investigation (equal); Methodology (equal); Writing – original draft (equal). **Yanyan Xu:** Data curation (equal); Formal analysis (equal); Investigation (equal); Methodology (equal); Writing – review & editing (equal). **Chang Cao:** Data curation (equal); Formal analysis (equal); Visualization (equal); Writing – review & editing (equal). **Bo Wang:** Data curation (equal); Investigation (equal); Visualization (equal). **Jiacheng Guo:** Data curation (equal); Formal analysis (equal); Investigation (equal). **Zhen Qin:** Formal analysis (equal). **Yongzheng Lu:** Formal analysis (equal). **Jianchao Zhang:** Formal analysis (equal). **Li Zhang:** Formal analysis (equal). **Wei Wang:** Formal analysis (equal). **Jinying Zhang:** Funding acquisition (equal); Project administration (equal); Supervision (equal); Writing – review & editing (equal). **Junnan Tang:** Conceptualization (equal); Project administration (equal); Resources (equal); Writing – review & editing (equal).

## Supporting information

Figure S1

Figure S2

Figure S3

Table S1

Table S2

## Data Availability

The data that support the findings of this study are available from the corresponding author upon reasonable request.

## References

[1] Hofmann R , James SK , Jernberg T , et al. Oxygen therapy in suspected acute myocardial infarction. N Engl J Med. 2017;377(13):1240‐1249. 28844200 10.1056/NEJMoa1706222

[2] World Health Organization . The top 10 causes of death. http://www.who.int/mediacentre/factsheets/fs310/en/ Accessed September 25, 2018.

[3] Kura B , Szeiffova Bacova B , Kalocayova B , Sykora M , Slezak J . Oxidative stress‐responsive microRNAs in heart injury. Int J Mol Sci. 2020;21(1):358. 10.3390/ijms21010358 PMC6981696 31948131

[4] Braunwald E . The war against heart failure: the Lancet lecture. Lancet. 2015;385(9970):812‐824. 25467564 10.1016/S0140-6736(14)61889-4

[5] Silvestre JS , Smadja DM , Lévy BI . Postischemic revascularization: from cellular and molecular mechanisms to clinical applications. Physiol Rev. 2013;93:1743‐1802. 24137021 10.1152/physrev.00006.2013

[6] Epelman S , Lavine KJ , Beaudin AE , et al. Embryonic and adult‐derived resident cardiac macrophages are maintained through distinct mechanisms at steady state and during inflammation. Immunity. 2014;40:91‐104. 24439267 10.1016/j.immuni.2013.11.019 PMC3923301

[7] Dutta P , Nahrendorf M . Monocytes in myocardial infarction. Arterioscler Thromb Vasc Biol. 2015;35:1066‐1070. 25792449 10.1161/ATVBAHA.114.304652 PMC4409536

[8] Nahrendorf M , Swirski FK , Aikawa E , et al. The healing myocardium sequentially mobilizes two monocyte subsets with divergent and complementary functions. J Exp Med. 2007;204:3037‐3047. 18025128 10.1084/jem.20070885 PMC2118517

[9] Swirski FK , Nahrendorf M , Etzrodt M , et al. Identification of splenic reservoir monocytes and their deployment to inflammatory sites. Science. 2009;325:612‐616. 19644120 10.1126/science.1175202 PMC2803111

[10] Bajpai G , Bredemeyer A , Li W , et al. Tissue resident CCR2‐ and CCR2+ cardiac macrophages differentially orchestrate monocyte recruitment and fate specification following myocardial injury. Circ Res. 2019;124:263‐278. 30582448 10.1161/CIRCRESAHA.118.314028 PMC6626616

[11] Vilahur G , Badimon L . Ischemia/reperfusion activates myocardial innate immune response: the key role of the toll‐like receptor. Front Physiol. 2014;5:496. 25566092 10.3389/fphys.2014.00496 PMC4270170

[12] Chen B , Huang S , Su Y , et al. Macrophage Smad3 protects the infarcted heart, stimulating phagocytosis and regulating inflammation. Circ Res. 2019;125(1):55‐70. 31092129 10.1161/CIRCRESAHA.119.315069 PMC6681442

[13] Frangogiannis NG . Emerging roles for macrophages in cardiac injury: cytoprotection, repair, and regeneration. J Clin Invest. 2015;125(8):2927‐2930. 26214519 10.1172/JCI83191 PMC4563767

[14] Torrieri G , Fontana F , Figueiredo P , et al. Dual‐peptide functionalized acetalated dextran‐based nanoparticles for sequential targeting of macrophages during myocardial infarction. Nanoscale. 2020;12(4):2350‐2358. 31930241 10.1039/c9nr09934d

[15] Nahrendorf M , Swirski FK . Monocyte and macrophage heterogeneity in the heart. Circ Res. 2013;112(12):1624‐1633. 23743228 10.1161/CIRCRESAHA.113.300890 PMC3753681

[16] Zhang Y , Liu Y , Liu H , Tang WH . Exosomes: biogenesis, biologic function and clinical potential. Cell Biosci. 2019;9:19. 30815248 10.1186/s13578-019-0282-2 PMC6377728

[17] Kalluri R , LeBleu VS . The biology, function, and biomedical applications of exosomes. Science. 2020;367(6478):eaau6977. 32029601 10.1126/science.aau6977 PMC7717626

[18] Vicencio JM , Yellon DM , Sivaraman V , et al. Plasma exosomes protect the myocardium from ischemia‐reperfusion injury. J Am Coll Cardiol. 2015;65(15):1525‐1536. 25881934 10.1016/j.jacc.2015.02.026

[19] Wang Y , Liu J , Ma J , et al. Exosomal circRNAs: biogenesis, effect and application in human diseases. Mol Cancer. 2019;18(1):116. 31277663 10.1186/s12943-019-1041-z PMC6610963

[20] Qian M , Wang S , Guo X , et al. Hypoxic glioma‐derived exosomes deliver microRNA‐1246 to induce M2 macrophage polarization by targeting TERF2IP via the STAT3 and NF‐κB pathways. Oncogene. 2020;39(2):428‐442. 31485019 10.1038/s41388-019-0996-y

[21] Singla DK , Johnson TA , Tavakoli DZ . Exosome treatment enhances anti‐inflammatory M2 macrophages and reduces inflammation‐induced pyroptosis in doxorubicin‐induced cardiomyopathy. Cells. 2019;8(10):1224. 10.3390/cells8101224 PMC6830113 31600901

[22] Almeida Paiva R , Martins‐Marques T , Jesus K , et al. Ischaemia alters the effects of cardiomyocyte‐derived extracellular vesicles on macrophage activation. J Cell Mol Med. 2019;23(2):1137‐1151. 30516028 10.1111/jcmm.14014 PMC6349194

[23] Ribeiro‐Rodrigues TM , Laundos TL , Pereira‐Carvalho R , et al. Exosomes secreted by cardiomyocytes subjected to ischaemia promote cardiac angiogenesis. Cardiovasc Res. 2017;113(11):1338‐1350. 28859292 10.1093/cvr/cvx118

[24] Ibanez B , James S , Agewall S , et al. 2017 ESC Guidelines for the management of acute myocardial infarction in patients presenting with ST‐segment elevation: The Task Force for the management of acute myocardial infarction in patients presenting with ST‐segment elevation of the European Society of Cardiology (ESC). Eur Heart J. 2018;39(2):119‐177. 28886621 10.1093/eurheartj/ehx393

[25] Théry C , Amigorena S , Raposo G , Clayton A . Isolation and characterization of exosomes from cell culture supernatants and biological fluids. Curr Protoc Cell Biol. 2006;Chapter 3:Unit 3.22. 10.1002/0471143030.cb0322s30 18228490

[26] Rezaeian AH , Li CF , Wu CY , et al. A hypoxia‐responsive TRAF6‐ATM‐H2AX signalling axis promotes HIF1α activation, tumorigenesis and metastasis. Nat Cell Biol. 2017;19(1):38‐51. 27918549 10.1038/ncb3445 PMC5441459

[27] Dabral S , Muecke C , Valasarajan C , et al. A RASSF1A‐HIF1α loop drives Warburg effect in cancer and pulmonary hypertension. Nat Commun. 2019;10(1):2130. 31086178 10.1038/s41467-019-10044-z PMC6513860

[28] Kumar A , Deep G . Hypoxia in tumor microenvironment regulates exosome biogenesis: Molecular mechanisms and translational opportunities. Cancer Lett. 2020;479:23‐30. 32201202 10.1016/j.canlet.2020.03.017

[29] Zhang Z , Tang J , Cui X , et al. New insights and novel therapeutic potentials for macrophages in myocardial infarction. Inflammation. 2021;44(5):1696‐1712. 33866463 10.1007/s10753-021-01467-2 PMC8460536

[30] Liu CL , Zhang X , Liu J , et al. Na^+^‐H^+^ exchanger 1 determines atherosclerotic lesion acidification and promotes atherogenesis. Nat Commun. 2019;10(1):3978. 31484936 10.1038/s41467-019-11983-3 PMC6726618

[31] Wang P , Wang H , Huang Q , et al. Exosomes from M1‐polarized macrophages enhance paclitaxel antitumor activity by activating macrophages‐mediated inflammation. Theranostics. 2019;9(6):1714‐1727. 31037133 10.7150/thno.30716 PMC6485189

[32] Jenkins SJ , Ruckerl D , Thomas GD , et al. IL‐4 directly signals tissue‐resident macrophages to proliferate beyond homeostatic levels controlled by CSF‐1. J Exp Med. 2013;210(11):2477‐2491. 24101381 10.1084/jem.20121999 PMC3804948

[33] Martinez FO , Sica A , Mantovani A , Locati M . Macrophage activation and polarization. Front Biosci. 2008;13:453‐461. 17981560 10.2741/2692

[34] Copple BL . Hypoxia stimulates hepatocyte epithelial to mesenchymal transition by hypoxia‐inducible factor and transforming growth factor‐beta‐dependent mechanisms. Liver Int. 2010;30(5):669‐682. 20158611 10.1111/j.1478-3231.2010.02205.x PMC3111074

[35] Shiraishi M , Shintani Y , Shintani Y , et al. Alternatively activated macrophages determine repair of the infarcted adult murine heart. J Clin Invest. 2016;126(6):2151‐2166. 27140396 10.1172/JCI85782 PMC4887176

[36] Su T , Huang K , Ma H , et al. Platelet‐inspired nanocells for targeted heart repair after ischemia/reperfusion injury. Adv Funct Mater. 2019;29(4):1803567. 32256277 10.1002/adfm.201803567 PMC7111457

[37] Su T , Huang K , Daniele MA , et al. Cardiac stem cell patch integrated with microengineered blood vessels promotes cardiomyocyte proliferation and neovascularization after acute myocardial infarction. ACS Appl Mater Interfaces. 2018;10(39):33088‐33096. 30188113 10.1021/acsami.8b13571 PMC6376980

[38] Tang J , Vandergriff A , Wang Z , et al. A regenerative cardiac patch formed by spray painting of biomaterials onto the heart. Tissue Eng Part C Methods. 2017;23(3):146‐155. 28068869 10.1089/ten.tec.2016.0492 PMC5367912

[39] Mihalko E , Huang K , Sproul E , et al. Targeted treatment of ischemic and fibrotic complications of myocardial infarction using a dual‐delivery microgel therapeutic. ACS Nano. 2018;12(8):7826‐7837. 30016078 10.1021/acsnano.8b01977

[40] Tang J , Cui X , Zhang Z , et al. Injection‐free delivery of MSC‐derived extracellular vesicles for myocardial infarction therapeutics. Adv Healthc Mater. 2021:e2100312. 34310068 10.1002/adhm.202100312

