# Supplementary figures and images for: Exosomes as a messager to regulate the crosstalk between macrophages and cardiomyocytes under hypoxia conditions
Source: J Cell Mol Med. 2022 Jan 28;26(5):1486–500. doi: 10.1111/jcmm.17162 (PMC8899199; doi:10.1111/jcmm.17162)

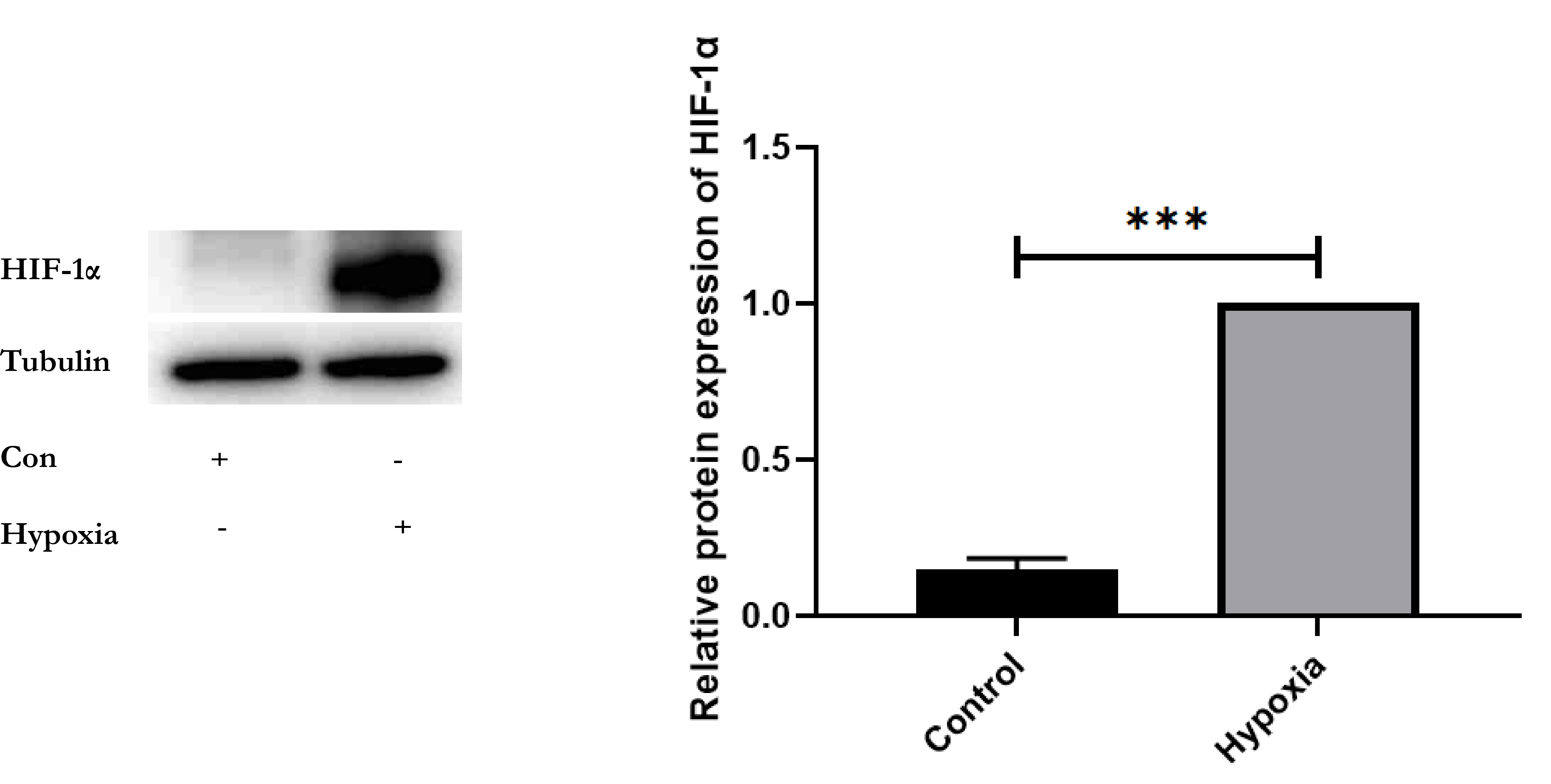

Supplement: Supplementary file 1 — Figure S1 [file JCMM-26-1486-s004.jpg]

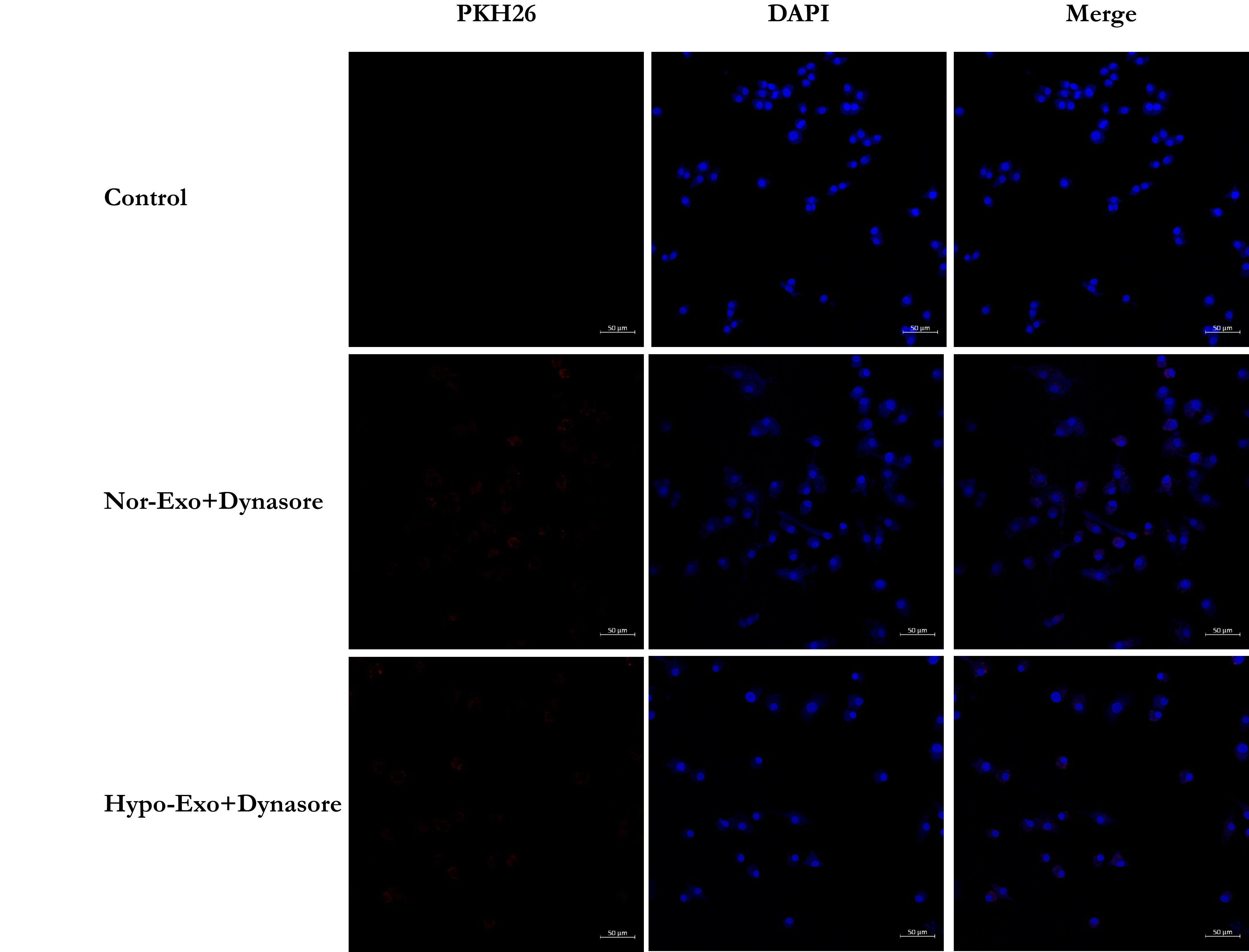

Supplement: Supplementary file 2 — Figure S2 [file JCMM-26-1486-s002.jpg]

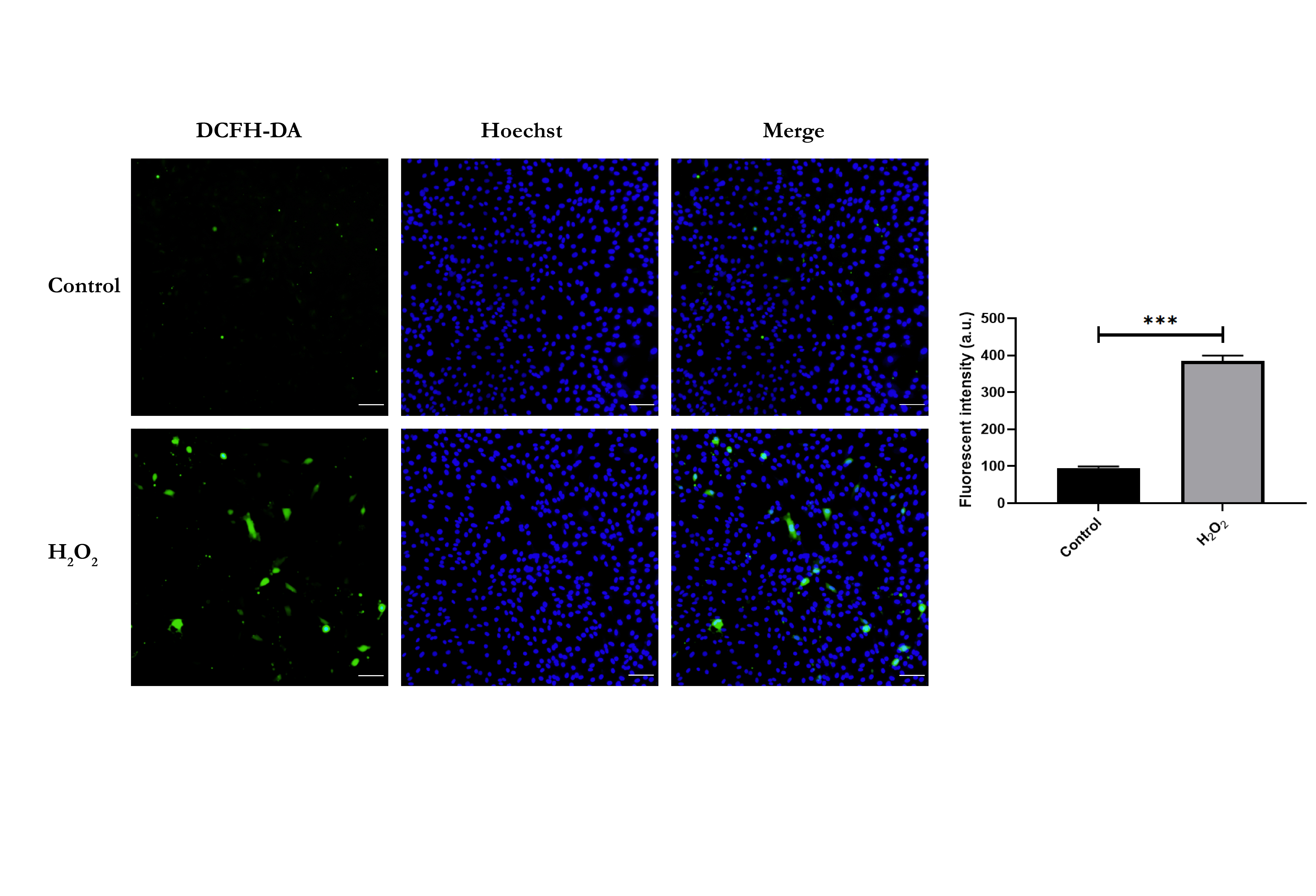

Supplement: Supplementary file 3 — Figure S3 [file JCMM-26-1486-s003.jpg]
