# Supplementary material for: Exosomes as a messager to regulate the crosstalk between macrophages and cardiomyocytes under hypoxia conditions
Source: J Cell Mol Med. 2022 Jan 28;26(5):1486–500. doi: 10.1111/jcmm.17162 (PMC8899199; doi:10.1111/jcmm.17162)
Supplement: Supplementary file 4 — Table S1 [file JCMM-26-1486-s005.docx]

| Characteristics | Control (N=5) | AMI (N=5) | *P* |
| --- | --- | --- | --- |
| Age (year)  Male (%)  Hypertension (%)  Smoker (%)  Drinker (%)  Glucose (mmol/L)  Cholesterol (mmol/L)  Triglyceride (mmol/L)  HDL (mmol/L)  LDL (mmol/L)  cTnT (ng/ml)  CK-MB (U/L)  CCB (%)  Spironolactone (%)  β-blocker (%)  Statin (%)  ACEI&ARB (%)  Aspirin (%) | 56.00±10.44  60  100  40  20  4.08±0.86  4.67±0.74  3.33±3.27  0.97±0.27  2.88±0.78  0.01±0.00  15.00±5.48  60  0  40  20  100  20 | 51.20±9.884  60  100  40  20  5.40±0.53  4.39±1.06  1.76±0.38  1.09±0.19  2.91±1.01  1.93±0.16  103.20±108.60  20  80  60  100  100  100 | 0.477  >0.999  >0.999  >0.999  >0.999  0.227  0.639  0.317  0.418  0.958  <0.001  0.107  0.524  0.048  >0.999  0.048  >0.999  0.048 |

Supplementary Table 1. Baseline characteristics of the enrolled patients.
