# Supplementary material for: Exosomes as a messager to regulate the crosstalk between macrophages and cardiomyocytes under hypoxia conditions
Source: J Cell Mol Med. 2022 Jan 28;26(5):1486–500. doi: 10.1111/jcmm.17162 (PMC8899199; doi:10.1111/jcmm.17162)
Supplement: Supplementary file 5 — Table S2 [file JCMM-26-1486-s001.docx]

Table S2. Sequences of primers used for real-time PCR analysis.

| Genes | Forward primers 5'-3' | Reversed primers 5'-3' |
| --- | --- | --- |
| Mouse GAPDH | CCTCGTCCCGTAGACAAAATG | TGAGGTCAATGAAGGGGTCGT |
| Mouse iNOS | CAACAGGAACCTACCAGCTCACT | AGCCTGAAGTCATGTTTGCCG |
| Mouse TNF-α | GTGCCTATGTCTCAGCCTCTTCTC | GTTTGTGAGTGTGAGGGTCTGG |
| Mouse IL-1β | ACAGGCTCCGAGATGAACAAC | GTGGGTGTGCCGTCTTTCAT |
| Mouse IL-6 | CCCCAATTTCCAATGCTCTCC | CGCACTAGGTTTGCCGAGTA |
| Mouse MCP-1 | GCAGGTCCCTGTCATGCTTCT | TGTCTGGACCCATTCCTTCTTG |
| Mouse Arg-1 | CTGGGGATTGGCAAGGTGAT | CAGCCCGTCGACATCAAAG |
| Mouse Fizz-1 | CTGCTACTGGGTGTGCTTGT | GCAGTGGTCCAGTCAACGAG |
| Mouse Ym-1 | TGGAGGATGGAAGTTTGGACC | TCAATGATTCCTGCTCCTGTGG |
| Mouse IL-10 | TTTAAGGGTTACTTGGGTTGCC | AATGCTCCTTGATTTCTGGGC |
| Mouse TGF-β | GCTGAACCAAGGAGACGGAATA | GGCTGATCCCGTTGATTTCC |
